# Trace Identification and Visualization of Multiple Benzimidazole Pesticide Residues on *Toona sinensis* Leaves Using Terahertz Imaging Combined with Deep Learning

**DOI:** 10.3390/ijms22073425

**Published:** 2021-03-26

**Authors:** Pengcheng Nie, Fangfang Qu, Lei Lin, Yong He, Xuping Feng, Liang Yang, Huaqi Gao, Lihua Zhao, Lingxia Huang

**Affiliations:** 1 College of Biosystems Engineering and Food Science, Zhejiang University, Hangzhou 310058, China; npc2012@zju.edu.cn (P.N.); ffqu@zju.edu.cn (F.Q.); linlei2016@zju.edu.cn (L.L.); zjuheyong@sina.com (Y.H.); pimmmx@163.com (X.F.); 2 Key Laboratory of Spectroscopy Sensing, Ministry of Agriculture and Rural Affairs, Hangzhou 310058, China; 3 State Key Laboratory of Modern Optical Instrumentation, Zhejiang University, Hangzhou 310027, China; 4 College of Animal Sciences, Zhejiang University, Hangzhou 310058, China; l_yang@zju.edu.cn (L.Y.); 3170100572@zju.edu.cn (H.G.); 5 Huzhou Agricultural Science and Technology Development Centre, Huzhou 313028, China; zlh98304025@sina.com

**Keywords:** pesticide residues, terahertz imaging, deep learning, image visualization, food safety

## Abstract

Molecular spectroscopy has been widely used to identify pesticides. The main limitation of this approach is the difficulty of identifying pesticides with similar molecular structures. When these pesticide residues are in trace and mixed states in plants, it poses great challenges for practical identification. This study proposed a state-of-the-art method for the rapid identification of trace (10 mg·L^−1^) and multiple similar benzimidazole pesticide residues on the surface of *Toona sinensis* leaves, mainly including benzoyl (BNL), carbendazim (BCM), thiabendazole (TBZ), and their mixtures. The new method combines high-throughput terahertz (THz) imaging technology with a deep learning framework. To further improve the model reliability beyond the THz fingerprint peaks (BNL: 0.70, 1.07, 2.20 THz; BCM: 1.16, 1.35, 2.32 THz; TBZ: 0.92, 1.24, 1.66, 1.95, 2.58 THz), we extracted the absorption spectra in frequencies of 0.2–2.2 THz from images as the input to the deep convolution neural network (DCNN). Compared with fuzzy Sammon clustering and four back-propagation neural network (BPNN) models (TrainCGB, TrainCGF, TrainCGP, and TrainRP), DCNN achieved the highest prediction accuracies of 100%, 94.51%, 96.26%, 94.64%, 98.81%, 94.90%, 96.17%, and 96.99% for the control check group, BNL, BCM, TBZ, BNL + BCM, BNL + TBZ, BCM + TBZ, and BNL + BCM + TBZ, respectively. Taking advantage of THz imaging and DCNN, the image visualization of pesticide distribution and residue types on leaves was realized simultaneously. The results demonstrated that THz imaging and deep learning can be potentially adopted for rapid-sensing detection of trace multi-residues on leaf surfaces, which is of great significance for agriculture and food safety.

## 1. Introduction

Benzimidazole (BZM) pesticides are widely used as agricultural insecticides and fungicides to control parasitic infections as well as crop diseases [1,2]. However, the overuse, misuse, or mixed-use of multiple BZM pesticides is potentially hazardous to human health, and even to the environment and ecosystems [3]. In particular, the mixed-use of BZM pesticides poses a challenge in the qualitative detection of multiple residues, because the BZM pesticide molecules have a similar functional group structure to that of benzimidazole and similar spectral vibration information [4]. Consequently, multi-residue detection has attracted wide attention and deserves further investigation, since it enables the identification of many pesticide residues [5]. To detect pesticide multi-residues in food matrices, several conventional laboratory analytical methods have been widely used, such as high-performance liquid chromatography (HPLC), gas chromatography mass spectroscopy (GC-MS), and liquid chromatography mass spectroscopy (LC-MS) [6]. These techniques offer a simultaneous high-throughput analysis of different pesticide residues; however, they are time-consuming and the required sample pre-treatments can be complex [7]. Additionally, typical spectrometry methods, including fluorescence spectroscopy (FS) and Raman spectroscopy (RS), have also been used to detect pesticide residues in food matrices [8]. Although these methods are fast and non-destructive, FS and RS fail to provide high sensitivity of multicomponent detection due to the interference of the fluorescence signal [9]. A reliable and dedicated method is thus needed to detect multiple BZM residues in leafy vegetables.

Terahertz (THz) imaging has become a novel technology to detect multiple pesticide residues [10]. The THz absorption peaks of various BZM pesticides can be used as unique signatures to distinguish each analyte in the mixture and thereby identify multiple pesticides [11,12]. The low-energy level of THz radiation does not pose significant safety risks to living organisms, making THz imaging a safe technology used in biological applications [13]. Additionally, the spectral or imaging data collected by THz imaging are abundant, including time domain, frequency domain, and amplitude information, as well as phase information that cannot be obtained using other sensors [14]. With ongoing advances in the development of THz systems, THz spectroscopy and imaging have been successfully used for pesticide determination in agricultural products [15,16]. However, these studies detected each component separately and prepared a high concentration of the component due to the limitations of detection. Detecting pesticide residues in food using THz technology would demand combined results from THz fingerprints and data analyses by machine learning [17]. THz technology and machine learning were used in pesticide detection, mainly through the following steps: (1) preprocessing of data by de-noising or baseline correction; (2) selection of fingerprint peaks; and (3) development of predictive models for new data analysis [18]. Compared to traditional chemometric-based classification techniques, deep learning algorithms could enable better insights into complex data characteristics at high levels of abstraction [19]. In addition, these algorithms avoid the problems of over-optimization and over-fitting [20]. The advantages of deep learning have motivated us to use this approach to classify and identify multiple pesticide residues on leaves [21].

In this study, we aimed to explore the validity of simultaneous and rapid identification of trace BZM multi-residues (benzoyl (BNL), carbendazim (BCM), and thiabendazole (TBZ)) on leaves of *Toona sinensis* (*T. sinensis*) using THz imaging and deep learning. In this experiment, the THz spectra (0.2–2.2 THz) of the tested leaves (treated with single- or multi-components of BNL, BCM, and TBZ with a concentration of 10 mg·L^−1^) were extracted from the regions of interest (ROIs) in each THz image. This work mainly focused on (1) characterizing the THZ fingerprint peaks of BNL, BCM, and TBZ, as well as simulating their molecular dynamics by solid density functional theory (DFT); (2) revealing the feasibility of classifying trace multi-residues on leaves based on their fingerprints and full spectra; (3) identifying the trace multi-residues on leaves rapidly and simultaneously using a framework combining THz imaging and a deep convolution neural network (DCNN); and (4) visualizing the multi-residue types and distributions of pesticides on leaves using the proposed framework. To the best of our knowledge, this is the first time that THz imaging and deep learning have been used for the rapid and simultaneous detection of trace BZM multi-residues on fresh leaves.

## 2. Results and Discussion

### 2.1. Molecular Dynamic Simulation of Pesticides

**Figure 1:**
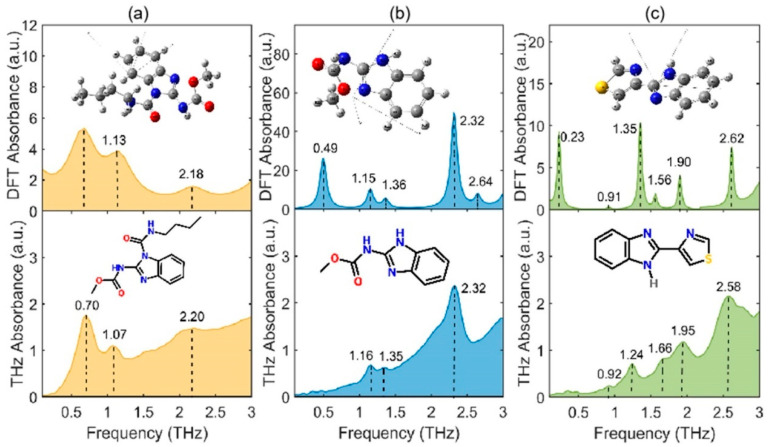
Comparison of terahertz (THz) and density functional theory (DFT) spectra of benzoyl (BNL), carbendazim (BCM), and thiabendazole (TBZ). (**a**) Spectra of BNL molecule. (**b**) Spectra of BCM molecule. (**c**) Spectra of TBZ molecule.

Figure 1 depicts the measured and simulated spectra of BNL, BCM, and TBZ. The THz spectra show that there are several characteristic absorption peaks for these three BZM pesticides in the frequency range of 0.1–3.0 THz. In order to explain the mechanism behind the formation of these absorption peaks, quantum chemistry calculations of the pesticide molecules were carried out using Gaussian 2016 software (Gaussian Inc., Wallingford, CT, USA). The hybrid functional model of B3LYP Becke with the 6−311G basis set (Lee-Yang-Parr functional B3LYP/6−311G) was used to optimize the single molecular structure of each pesticide and simulate its DFT spectrum. The molecular vibration modes of each peak were determined by comparing the experimental and theoretical spectra.

As shown in Figure 1a, the THz absorption peaks of BNL were measured at 0.70, 1.07, and 2.20 THz and could be matched with the simulated peaks at 0.66, 1.13, and 2.18 THz, respectively. According to the DFT simulated results (as listed in Table 1), the absorption peak of BNL at 0.70 THz could be explained by the annular respiratory vibration of the whole BNL molecule. The peak at 1.07 THz was generated by the out-plane bending vibration of the single bond C-N (the 14C atom in methylcarbamate and 13N atom in the benzimidazole group). The peak at 2.20 THz was formed by the in-plane bending vibration of the single bond C-O (the 36C and 35O atoms in N-butylcarbamyl). To calculate the results for BCM (Figure 1b), the measured THz peaks (1.16, 1.35, and 2.32 THz) were in reliable agreement with the DFT simulated peaks (1.15, 1.36, and 2.32 THz), except for the slight shift in frequency and two missing peaks (0.49 and 2.64 THz). The peak at 1.16 THz was caused by the interaction of the in-plane torsional vibration of the acylamino groups, composed of C-N (17C and 15N) and C-O (17C and 19O). The peak at 1.35 THz was formed by the in-plane torsional vibration of the benzimidazole functional group. The peak at 2.32 THz was generated by the in-plane torsional vibration of the C-O (19C and 20O) carboxylic ester. The two peaks missing at 0.49 and 2.64 THz may have been covered by the noise of the THz time-domain spectroscopy (THz-TDS). For TBZ, the measured spectrum showed five peaks at 0.92, 1.24, 1.66, 1.95, and 2.58 THz. The matched DFT results (Figure 1c) showed six peaks at 0.23, 0.91, 1.35, 1.56, 1.90, and 2.62 THz. The peak missing at 0.23 THz may also have been obscured due to noise. The peak at 0.92 THz was formed by the out-plane respiratory vibration of the benzimidazole ring and aromatic heterocycle. The peak at 1.24 THz was formed by the out-plane respiratory vibration of the benzimidazole ring. The peaks at 1.66 and 1.95 THz were generated by the in-plane stretching vibration of C-C (the 7C atom of the benzimidazole group and the 15C of the aromatic heterocyclic compound). The peak at 2.62 THz was formed by the interaction of the in-plane bending vibrations of C-H (3C and 8H, 6C and 11H) in the benzimidazole group.

### 2.2. Spectral Analysis of Multiple BZM Pesticide Mixtures

**Figure 2:**
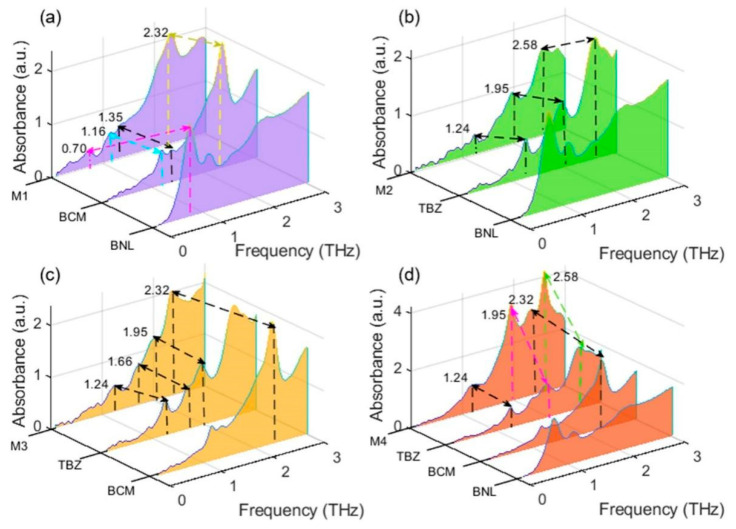
Spectral characteristic analysis of multiple pesticide mixtures. (**a**) Peak analysis of the benzoyl (BNL) and carbendazim (BCM) mixture (M1). (**b**) Peak analysis of the BNL and thiabendazole (TBZ) mixture (M2). (**c**) Peak analysis of the BCM and TBZ mixture (M3). (**d**) Peak analysis of the BNL, BCM, and TBZ mixture (M4).

In order to characterize the fingerprints of pesticide mixtures, the spectral absorption peaks of the multiple pesticide mixtures were compared and analyzed. As shown in Figure 2a, four absorption peaks at 0.70, 1.16, 1.35, and 2.32 THz were found in the spectrum of M1 (the mixture of BNL and BCM). Among them, the three peaks with higher frequencies and intensities originated from BCM. Only one peak (0.70 THz) originated from BNL, contributing a weak peak to the spectrum of M1. The three peaks (1.24, 1.95, and 2.58 THz) of M2 (mixture of BNL and TBZ) all came from TBZ and no BNL peak was shown in the spectrum of M2 (as shown in Figure 2b). For the four peaks shown in the spectrum of M3 (mixture of BCM and TBZ), one peak (2.32 THz) originated from BCM and three peaks (1.24, 1.66, and 1.95 THz) came from TBZ. Figure 2d demonstrates that none of the BNL characteristic peaks appeared in the spectrum of M4 (mixture of BNL, BCM, and TBZ), in which one peak at 2.32 THz originated from BCM and three peaks at 1.24, 1.95, and 2.58 THz originated from TBZ. These results showed that the peaks of BNL were almost invisible in the spectra of mixtures. The reason for this is that BNL is unstable and degrades quickly to BCM. In this respect, the bactericidal effect of BNL is due to its main metabolite BCM. TBZ was stable and its residues remained in plant-derived food for a long time. This compound provided the most significant characteristic peaks in the mixture spectrum.

By comparing and analyzing the mixture spectra in Figure 2a–d, the peak at 2.32 THz can be used as a fingerprint to identify the presence of BCM and the peak at 1.24 and 1.95 THz can be used to identify the presence of TBZ. As for BNL, the peak at 0.70 THz was chosen as its fingerprint because of its stronger absorption intensity compared with that of the other two peaks (1.07 and 2.20 THz). Therefore, the four absorption peaks at 0.70, 1.24, 1.95, and 2.32 THz were selected as the fingerprints of the three BZM pesticides. According to these specific fingerprints, the compositions, structures, and concentrations of the components contained in mixtures can be determined by analyzing the origins and intensities of the absorption peaks. This is of great significance in analyzing pesticide mixtures containing unspecified components at unknown concentrations.

### 2.3. Clustering Analysis Based on Absorption Peaks and Full Spectra

**Figure 3:**
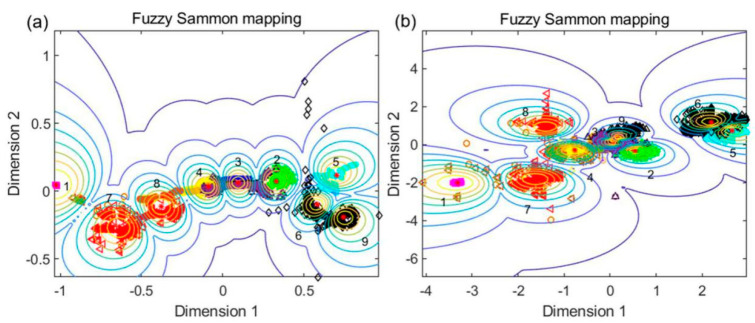
Clustering results of the nine classes of spectral data. (**a**) Clustering results based on fingerprints. (**b**) Clustering results based on full spectra.

Nine classes of absorbance spectra extracted from the THz images were clustered to explore structures in the data. Figure 3 depicts the structures, cluster centers, and space trajectories of the fuzzy Sammon mapping results. The parameters were set to number of maximum iterations, t = 20; iteration step size, s = 1; and weighting exponent, m = 2. The cluster numbers corresponding to background (BG), control check (CK), BNL, BCM, TBZ, M1, M2, M3, and M4 were labeled 1 to 9, respectively. The classification accuracies of the clustering results were calculated based on the selected fingerprints (0.70, 1.24, 1.95, and 2.32 THz) and the full spectra (0.2–2.2 THz) and were 69.49% and 70.58%, respectively. The mapping distances based on the full spectra (Figure 3b) were greater and the clusters were more compact than those based on the selected four fingerprints (Figure 3a), verifying that the results based on the full spectra were slightly more accurate. The data points were more concentrated in the ranges of clusters 1–5 and scattered in the ranges of clusters 6–9, illustrating that the classification ability decreased with an increase in the number of mixed pesticides. In addition, the fingerprint peaks of the trace pesticides were covered by the signals of the leaves. Therefore, it is better to identify the types of pesticide residues in leaves using the full spectra rather than characteristic fingerprints. The fuzzy Sammon mapping algorithm is a useful computational intelligence tool in user-guided clustering visualization and shows the feasibility of identifying different pesticide residue types in leaves. However, its identification results are unsatisfactory. More qualified methods, such as deep neural network models, should be used to accurately identify the different types of pesticide residues in leaves.

### 2.4. Identification of Multiple Pesticide Residues on T. sinensis Leaves

**Figure 4:**
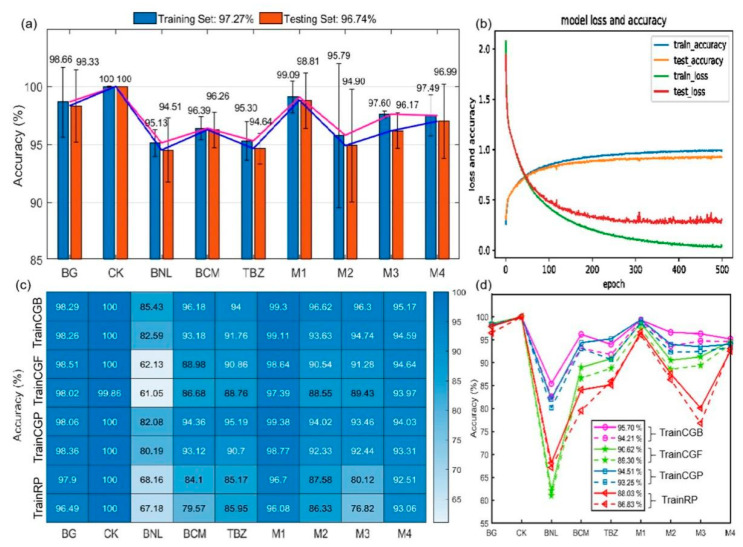
Modeling performance of the deep convolution neural network (DCNN) and back-propagation neural network (BPNN). (**a**) Accuracy of the DCNN model. (**b**) Loss and accuracy of DCNN during iteration. (**c**) Heatmap of BPNN based on learning functions of TrainCGB, TrainCGF, TrainCGP, and TrainRP. (**d**) Accuracy curves of BPNN models.

In order to accurately detect the trace and multiple BZM pesticide residues on *T. sinensis* leaves, the DCNN model was established based on the nine classes of spectra (0.2–2.2 THz) extracted from their THz images. In addition, four BPNN models (TrainCGB, TrainCGF, TrainCGP, and TrainRP) were established and compared with DCNN. Figure 4 displays the identification results of these five neural network models. The DCNN training and testing results for each category achieved highly satisfactory levels of accuracy (Figure 4a). Figure 4b plots the loss and accuracy of the DCNN model during the process of iteration. The accuracy of the DCNN model gradually stabilized after 250 iterations. Accordingly, the loss for the testing set gradually was reduced with a certain extent of fluctuation after 250 iterations, and the loss for training procedures stayed reduced even after 250 iterations. Compared with the testing set, the training set had higher accuracy and lower loss, proving the robustness of the DCNN model and the absence of over-fitting. In particular, the modeling accuracy of the CK samples without pesticide residues achieved 100% both for the training and testing sets. In contrast, DCNN achieved the lowest identification accuracy for leaf samples containing BNL residues, which might be due to the instability and degradability of BNL. As for the BG samples (no leaves or pesticides), the accuracy in training and testing did not reach 100%, which might be due to interference from instrument noise. The modeling accuracy for the samples with multiple pesticide residues (M1, M2, M3, and M4) was also excellent. For all nine classes of samples, the average identification accuracies of the training and testing sets were 97.27% and 96.74%, respectively. These results demonstrate that the DCNN model can be used as an effective tool to identify different pesticide residues on leaves.

Figure 4c,d show the heatmap and accuracy curves of the BPNN models established based on four learning functions (TrainCGB, TrainCGP, TrainCGF, and TrainRP). As can be seen from the heatmap, the four BPNN models obtained the highest accuracy for CK and the lowest accuracy for BNL, coinciding with those of the DCNN model. These results further illustrated the specificity of CK and BNL samples in the DCNN and BPNN models. In addition, these results validate the feasibility of identifying multiple pesticide residues on leaves using different neural network models. Figure 4d shows the accuracy curves, in which solid lines represent the training set and dotted lines represent the testing set. The accuracy curves were used to compare the properties among the four learning functions. The lowest BPNN results were obtained based on the TrainRP algorithm. The average accuracies of the training and testing set were 88.06% and 86.83%, respectively. In addition, the performances of the BPNN models established based on TrainCGB, TrainCGP, and TrainCGF were desirable. The average accuracy obtained from these networks ranged from 90.62% to 95.70% for the training set and from 89.30% to 94.21% for the testing set. The higher results of TrainCGB, TrainCGP, and TrainCGF might be due to their built-in oscillation functions, which prevented them from remaining at a local minimum. Although the model accuracy of BPNN was not as high as that of DCNN, all functions generated reliable performances. The modeling performance showed that DCNN was more desirable due to the feature of automatic extraction from high-dimensional data, as well as higher levels of robustness and accuracy.

### 2.5. Visualization of Pesticide Residue Distributions on T. sinensis Leaves

Eight *T. sinensis* leaves treated with different pesticide residues were selected to visualize the distribution of pesticide multi-residues. Among the neural network models, DCNN achieved the best accuracy of identification and, therefore, was used to determine which pesticide residues were present on the tested leaf samples. Spectral data in the frequency range of 0.2–2.2 THz were used as the input data for the DCNN model. The output value of the DCNN model was the predicted class index label of each pixel, which corresponded to a class of input spectrum.

**Figure 5:**
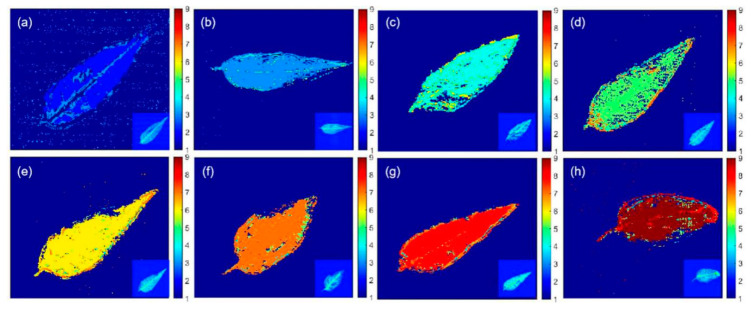
Deep convolution neural network (DCNN) visualization of *Toona sinensis* leaves with different pesticide residues. (**a**) Control check (CK) leaf without pesticide residues, (**b**) leaf with benzoyl (BNL) residue, (**c**) leaf with carbendazim (BCM) residue, (**d**) leaf with thiabendazole (TBZ) residue, (**e**) leaf with BNL and BCM residues, (**f**) leaf with BNL and TBZ residues, (**g**) leaf with BCM and TBZ residues, and (**h**) leaf with BNL, BCM, and TBZ residues.

Figure 5 shows the visualized THz images of the eight *T. sinensis* leaves. It clearly reveals the shape of the leaves and the spatial distribution of the pesticide residues on them. The predicted labels 1–9 represented the classes of BG, CK, BNL, BCM, TBZ, M1, M2, M3, and M4, respectively, which were represented by different colors. The distribution of multi-residues in the leaves were displayed by DCNN imaging visualization, which could not be observed with the naked eye in the original leaf samples. Although some error labels occurred in BG regions, which may be caused by interference from instrument noise, the leaf regions were perfectly presented in the visual images. Figure 5a displays the leaf image of the CK sample. The labeling results in each pixel were generally accurate, but there were some identification errors on the veins and edges of the leaf. As shown in Figure 5b–d, the images of leaves containing BNL, BCM, and TBZ pesticide residues, were highly representative of their class labels. Figure 5e,f show the distributions of M1 (BNL + BCM) and M2 (BNL + TBZ) multi-residues, respectively. Due to the instability of BNL, the colormaps in Figure 5e,f exhibited not only the majority of the information about the multi-residues of M1 and M2, but also the minority information of the single component pesticides, BCM and TBZ. As can be seen from Figure 5g, the imaging results from M3 (BCM + TBZ) were more desirable compared to those of M1 and M2. For M4 (BNL + BCM + TBZ), as shown in Figure 5h, the predicted labels were more complex. These results implied that with the increase in the number of pesticide components, the DCNN prediction accuracy for each pixel would decrease, posing a challenge to the detection of multiple pesticide residues. However, by counting the majority labels as final, the identification accuracy of the pesticide residue type was excellent.

## 3. Materials and Methods

### 3.1. Sample Preparation

#### 3.1.1. Preparation of BZM Pesticide Pellets

To characterize the absorption peaks of the pesticides and analyze the spectral features of their mixtures, solid-state pesticide samples were prepared to obtain their THz absorption spectra. Standards of BNL (C_14_H_18_N_4_O_3_), BCM (C_9_H_9_N_3_O_2_), and TBZ (C_10_H_6_N_2_S_2_) were purchased from Sigma-Aldrich (St. Louis, MO, USA). High-density polyethylene (HDPE; Sigma-Aldrich) was used as the binder when pressing pesticide samples into pellets, because it demonstrates weak absorption of THz radiation and, in general, has no effect on the location of pesticide absorption peaks. For single-component samples, BNL, BCM, and TBZ were each mixed with HDPE at a 1:1 ratio. Samples containing two components, such as BZL + BCM (M1), BZL + TBZ (M2), or BCM + TBZ (M3), were mixed with HDPE at a ratio of 1:1:2. A mixture of three pesticides, BNL + BCM + TBZ (M4), was mixed with HDPE at a ratio of 1:1:1:3. One sample with pesticide-free HDPE was used as the CK reference. The total mass of all the samples was 200 mg and HDPE accounted for half of the mixture content. The mixtures were homogenized in an agate mortar, sifted through a 200-mesh screen, and pressed into pellets under 16 tons of pressure for 4 min. The final prepared samples were disc-shaped pellets with diameters of 13 mm.

#### 3.1.2. Preparation of *Toona sinensis* Leaf Samples

The BZM solutions were dripped onto the surfaces of *T. sinensis* leaves to obtain their THz images and thus to qualitatively identify multiple BZM pesticide residues in fresh leaves. Fresh *T. sinensis* was purchased from the local organic vegetable market (Hangzhou, China) and cleaned with ethanol (Sigma-Aldrich) before preparing leaf samples. BZM pesticide solutions with concentrations of 10 mg·L^−1^ were prepared by mixing 1 mg of each type of the BZM pesticide standards in 100 mL of acetonitrile. Seven pesticide solutions were prepared, including three single-component solutions (BNL, BCM, and TBZ), three two-component solutions (BNL + BCM, BNL + TBZ, and BCM + TBZ), and one three-component solution (BNL + BCM + TBZ). In addition, 100 mL of pesticide-free acetonitrile solution was used for the CK group. After mixing evenly, 7 mL of each solution was dripped over the entire surface of *T. sinensis* leaves with a pipette. For each type of solution, 26 replicates were made to generate 208 leaf samples in total.

### 3.2. THz Data Acquisition and Extraction

#### 3.2.1. THz Spectra and Image Acquisition

THz spectra and imaging measurements were carried out using a THz time-domain spectroscopic system CCT−1800 (China Communication Technology Co., Ltd., Shenzhen, China). Both the pellet and leaf samples were placed in a sample chamber. The chamber was filled with dry nitrogen to prevent moisture from interfering with THz signals. The absorption spectra of the pesticide pellet samples were obtained using the transmission-scanning mode. In this mode, HDPE was used as the reference and the spectral resolution was 30 GHz over a range of 0.1–3 THz. All absorption spectra were the average results of five scans and five measurements. Before imaging, the pesticide-coated *T. sinensis* leaves were dried at room temperature to volatilize the acetonitrile. The leaves were then fixed flat on the sample shelf using transparent tape. Absorption images of the leaf samples were obtained using the transmission-imaging mode. In this mode, transparent tape was used as a reference, and the scanning frequency was 15 Hz over an image size of 250 × 250 pixels.

#### 3.2.2. Spectral Extraction from THz Images

For spectral extraction, THz images of the 208 leaf samples were divided into two sets. Among them, 200 THz images of the eight residue types (25 images of each type; one CK and seven pesticide residue types) were used to extract leaf spectra and identify the residues. To extract the spectra, an ROI (3 × 3 pixels) was used as a shift operator to scan the leaf area step by step. The spectrum of each pixel in the ROI was extracted, and the average value was taken to give the spectrum of the ROI. Accordingly, 100 spectra were extracted from the THz images of each leaf sample and, thus, 20,000 spectra (2500 for each of the eight residue types) were extracted from 200 leaves. In order to avoid segmentation of the leaf images to facilitate visualization, 2500 spectra of the tape background (BG) were extracted from a group of 25 images. Therefore, a total of 22,500 spectra from the nine classes (one BG and eight types of residue, including a CK) were extracted to establish and validate the network models. The remaining eight THz images (one for each type) were used to visualize the residue distribution on leaves. All the spectra of the eight THz images (62,500 for each) were extracted and input into the established network models to predict the class labels. Visualizations of the pesticide residue types on leaves were performed by reconstructing the 62,500 class labels into 250 × 250-pixel images. All extracted spectra were intercepted at frequencies of 0.2–2.2 THz from the original range of 0.1–3 THz to remove the spectral noise.

### 3.3. Neural Network Classification Models

**Figure 6:**
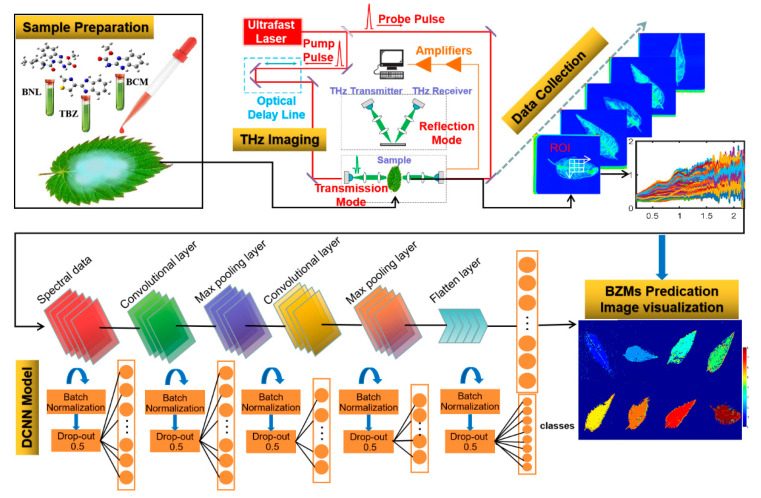
The main flow chart for detecting multiple benzimidazole (BZM) pesticide residues in leaves of *Toona sinensis* using terahertz (THz) imaging and deep learning.

Discrimination models, including a deep convolution neural network (DCNN) and four back-propagation neural network (BPNN) models, were used to identify the types of residues on *T. sinensis* leaves based on the THz fingerprint. Figure 6 shows the flow chart for the whole process. This process provides a feasible strategy to rapidly and accurately detect pesticide multi-residues on plant leaves.

#### 3.3.1. Deep Convolution Neural Network

In this paper, a DCNN model consisting of a convolution layer, pooling layer, and fully connected layer was used to identify multi-pesticide residues on leaves. There were two convolution modules and five fully connected layers in the DCNN framework to process nine classes (BG, CK, and seven types of pesticide residues) of input spectral data, of which 70% (15,750) of the data were used for training and 30% (6750) were used for testing. The convolution module contained a convolution layer and a maximum pooling layer. The number of convolution filters in the first and second convolution layers was set to 32 and 64, respectively. In order to learn the local information quickly and reduce the dimensions of the spectral data, the step size of the convolution kernel was set to 1. The kernel size of the convolution layers and the maximum pooling layer was set to 1 × 3. Rectified linear units (ReLUs) were used for the attribution of the non-linear properties of the decision mapping function [22]. Batch normalization (BN) was used before each full connection layer and after each convolution module to improve learning speed and reduce initialization requirements [23]. The Softmax function was used to process the full-connectivity data of the last layer by highlighting the maximum value and limiting the eigenvalues of other nerve units below the maximum value. The classified cross-entropy loss function was used to measure the distance of the probability distributions between the DCNN output labels and the real class labels [24]. An adaptive moment estimation algorithm was used to process the loss function. In order to prevent over-fitting during the training process, 30% of the training set was selected for verification and a drop-out method was used to further prevent over-fitting after batch normalization of all connection layers. The epoch number, learning speed, beta 1, and beta 2 were set to 500, 0.001, 0.9, and 0.99, respectively.

#### 3.3.2. Back-Propagation Neural Networks

In comparison with DCNN, four learning functions, including the Powell-Beale conjugate gradient algorithm (TrainCGB), the Fletcher-Reeves conjugate gradient algorithm (TrainCGF), the Polak-Ribiere conjugate gradient algorithm (TrainCGP), and Resilient Back-Propagation (TrainRP) were utilized for the back-propagation neural networks (BPNN) models [25]. To achieve a higher classification accuracy of a large amount of spectral data (15,750 spectra for training and 6750 for testing), four-layer networks were constructed, in which the input layer and the two hidden layers all consisted of 40 nodes and the output layer consisted of nine nodes. Links were used to connect the nodes of different layers. Two parameters (weight and bias) in each link were updated or estimated according to the four functions, TrainCGB, TrainCGF, TrainCGP, and TrainRP [26]. The learning rates of 0.1 were set by default, which determined the size of the weight updated at the end of each epoch, thereby influencing the rate of convergence. The number of epochs was set to 5000, which defined the maximum number of iterations for training. The learning goals were set to 0.0001, which determined the accuracy of the output results.

### 3.4. Data Analysis and Image Visualization

The fuzzy Sammon mapping method was used to visualize the spectral clustering results, which represented the separation tendencies or inter-pattern distances among different samples [27,28]. Heatmap analysis was used to visualize the accuracy of the identification models. Additionally, the distributions of different pesticide residues on *T. sinensis* leaves were visualized by restoring the class labels (predicted by DCNN) into two-dimensional images. THz spectral analysis, fuzzy Sammon mapping, heatmap analysis, and BPNN models were performed using Matlab 2018b (MathWorks, Natick, MA, USA). DCNN modeling was conducted using Python version 3.6.0.

## 4. Conclusions

In this study, a simple, rapid, and robust method was developed. This method combines THz imaging with deep learning to determine trace and multiple BZM pesticide residues on *T. sinensis* leaves. The THz absorption peaks of solid-state BZM pesticides (BNL, BCM, and TBZ) were monitored, and the mechanisms by which these absorption peaks formed were revealed theoretically by DFT simulation of the molecular dynamics. With a trace amount (10 mg·L^−1^) of pesticide solution dripped onto the surfaces of *T. sinensis* leaves, THz images of leaves were obtained and the absorption spectra (0.2–2.2 THz) were extracted from ROIs. The spectral characteristics of the absorption peaks were undetectable due to extremely low residue levels on the leaves; however, based on both the full spectra and the fingerprint peaks, fuzzy Sammon mapping clustering results demonstrated the feasibility of identifying different residue types. An advanced neural network model DCNN was established to identify a total of nine classes of spectra, including a reference background, CK group, and seven types of pesticide residue samples. As a representative algorithm of deep learning, DCNN has the ability to extract higher-order features from input information and has considerable superiority with respect to avoiding overfitting and good stability. Using this method, satisfactory accuracy in identifying different pesticide multi-residues were obtained and reliable multi-residue distributions were visualized. This study demonstrated that the method based on THz imaging and deep learning can be extremely useful to analyze trace multi-residue pesticides on leaves of *T. sinensis*. This qualitative analytical reference method may be used for the routine determination of pesticides in other green leafy vegetables to ensure food safety.

## Figures and Tables

**Table 1:** Peak assignments for BNL, BCM, and TBZ pesticide molecules. υ: stretching vibration, δ: bending vibration, oop: out-plane bending, ip: in-plane bending.

DFT Simulation (THz)	THz Experiment (THz)	Shift (THz)	Vibration Modes
benomyl			
0.66	0.70	−0.04	υring
1.13	1.07	0.05	δ(C-N)oop
2.18	2.20	−0.02	δ(C-O)ip
carbendazim			
0.49	-	-	δ(C-O)ip, δ(C-)ip
1.15	1.16	−0.01	δ(C-O)ip, δ(C-)ip
1.36	1.35	0.01	δring
2.32	2.32	-	δ(C-O)ip
2.64	-	-	δring
thiabendazole			
0.23	-	-	δ(C-C)ip
0.91	0.92	−0.01	δring
1.35	1.24	0.11	δring
1.56	1.66	−0.10	υ(C-C)ip
1.90	1.95	−0.05	δ(C-C)ip
2.62	2.58	0.04	δ(C-H)ip

## Data Availability

Not applicable.
